# Pharmacovigilance of Antitubercular Therapy in Tuberculosis

**DOI:** 10.7759/cureus.21915

**Published:** 2022-02-04

**Authors:** Mirunalini Ravichandran, Manju Rajaram, Malathi Munusamy

**Affiliations:** 1 Pharmacology, Jawaharlal Institute of Postgraduate Medical Education and Research, Puducherry, IND; 2 Pulmonary Medicine, Jawaharlal Institute of Postgraduate Medical Education and Research, Puducherry, IND; 3 Dermatology, Jawaharlal Institute of Postgraduate Medical Education and Research, Puducherry, IND

**Keywords:** severity, causality, adverse event, tuberculosis, antitubercular drugs, pharmacovigilance, adverse drug reaction

## Abstract

Background

Adverse drug reactions (ADRs) to tuberculosis (TB) drugs are a significant concern for medical professionals and health authorities. Adverse events due to drug-resistant TB (DRTB) treatment are among the most important reasons for treatment interruption.

Methods

This study was an observational study conducted among patients diagnosed with TB (pulmonary/extrapulmonary) receiving antitubercular therapy (ATT) (first line/second-line drugs) irrespective of their age and gender. The patients who consented to participate, registered under National Tuberculosis Elimination Program (NTEP), Puducherry, during the study period from March 2020 to December 2020, were included in the study. The demographic details were recorded from the treatment card, and the participants were provided a diary to note down the adverse events. They were asked to report over the phone or during their visits to treatment centers for the first two months. During the follow-up (irrespective of their treatment phase [intensive/continuation]), the patients were assessed for symptoms and signs of common adverse events. Any adverse events reported by the patient were also recorded and analyzed for causality and severity.

Results

During the study period, 219 patients were included, of which 92 patients (42%) presented with adverse events. Among the patients with ADRs, 56.5% were males and 43.5% were females. The females were found to be at more risk than males for adverse events with the OR 1.871 (95% CI: 1.066-3.284). GI system was the most common body system involved (39%), followed by musculoskeletal system and skin disorders (24% and 21%), respectively. Most of the adverse events were latent in nature (60.9%), followed by sub-acute onset (28.3%) and acute events (6.5%). Maximum adverse events reported were mild to moderate (71.8%), followed by severe (18.5%). Most of the events were probable in nature (41.3%), and the definite category was 25% as per Naranjo’s probability scale.

Conclusion

The current study shows the frequency of adverse events in patients receiving antitubercular drug therapy. The females were found to be at more risk than males for adverse events. It was found that the GI system was most affected as a known reaction to TB therapy followed by the musculoskeletal system. With more effective pharmacovigilance measures implementation, the adverse events being one of the factors for treatment interruption can be overcome.

## Introduction

Tuberculosis (TB) has become one of India's biggest public health challenges and one of the top ten causes of death worldwide. Globally, an estimated 10 million people fell ill with TB. About a quarter of the world's population is infected with Mycobacterium tuberculosis, and thus the lifetime risk of developing TB is estimated to be 5-10%. India is one of the eight high TB burden countries, constituting 87% of the TB cases [1,2].

Adverse drug reactions (ADRs) to TB drugs have become a significant area of concern for medical professionals and health authorities. Identification of the ADR profile of drugs can be helpful for prevention, early detection, and management of ADRs and to improve adherence to the therapy. It is essential to know the prevalence of various ADRs and their management. Therefore, identifying the causality and severity of ADRs is an essential step in ADR monitoring programs [3]. Studies conducted in India have shown the difference in the incidence of the ADRs to TB therapy, especially in particular groups like drug-resistant TB (DRTB), pediatric TB, and antitubercular therapy (ATT) with highly active antiretroviral therapy [4-7].

Adverse events due to DRTB treatment are among the most important reasons for treatment interruption [8]. Many studies in India have highlighted various ADRs to ATT [9-11]. However, with the changing trends in the management of TB, the guidelines are modified, and the changing regimens necessitate the need for updating the adverse drug events to the therapy. This reporting of ADRs could identify a new signal in the pharmacovigilance of ATT. Hence this study was planned to analyze ADR to ATT and associated risk factors among the patients registered under the district TB center of Puducherry.

## Materials and methods

This study was an observational study conducted from March 2020 to December 2020. The study was initiated after obtaining the ethics approval. The eligibility criteria for the study were the patients diagnosed with TB (microbiologically confirmed and clinically diagnosed), either pulmonary or extrapulmonary, started on ATT irrespective of their age and gender, and consenting to participate. Patients registered under the Revised National TB Control Programme (RNTCP) in the national TB registry of the Government Chest Clinic and Government Chest Hospital, Puducherry, were included in the study. Patients were included in the study after explaining and obtaining their written informed consent. The patients from the Government Chest Hospital were those admitted to the hospital who encountered an adverse event during the treatment period or admitted for any other reason having an adverse event to ATT. The demographic details were recorded from the treatment card. In order to study the adverse event to ATT, the participants were provided a diary and asked to note down the adverse effects experienced by them (noted either by the patient or by the caregiver, whoever was able to document the events) every day for two months. In between, if they develop any ADRs, they were asked to report. All participants were asked about the adverse effects experienced by them and verified the same with the diary during their follow-up visit (irrespective of their treatment phase: intensive/continuation) to the treatment centers. They were also assessed for symptoms and signs of common adverse effects. Any adverse effects reported by the patient were also recorded. The collected adverse events reports were documented and analyzed for causality and severity. ADRs were also classified based on WHO's System Organ Classes. The causality of ADRs was assessed by Naranjo's algorithmic scale [12]. The severity of the ADRs was assessed by the modified Hartwig and Siegel scale and graded as mild, moderate, and severe [13] and the onset as acute, subacute, and latent [14].

Data were expressed as mean ± SD and percentages. A Chi-squared test was applied to prove their statistical significance, with a p-value of <0.05, which was considered significant. In addition, clinical factors like age, BMI, gender, type of tuberculosis (drug-sensitive TB [DSTB]/DRTB), treatment regimen, and comorbid conditions were analyzed for their association with the ADR using multiple logistic regression. Analysis was carried out with SPSS version 15 for windows.

## Results

During the study period, 219 TB patients started on ATT (first-line/second-line drugs) were included, of which 92 (42%) patients presented with adverse events. The participants' demographic details as observed with adverse events and without adverse events are described in Table 1. The mean age was 45.48 ± 17.884, and BMI was found to be 19.45 ± 5.36 among those with adverse events. Similarly, among the patients with adverse events, 52 (56.5%) were males and 40 (43.5) were females. However, it was found that the females were at more risk than males for adverse events (i.e., 40 females with adverse events out of a total of 77 females vs. 52 males with adverse events out of 142 males) with the OR 1.871 (95% CI: 1.066-3.284). It was observed that 81 patients (40.1%) out of 202 on the DSTB regimen had developed adverse events. Among the 92 participants who developed adverse events, 81 (88%) were on the DSTB regimen compared to DRTB. Table 1, which describes the proportion of patients with and without adverse events based on their demographic and clinical characteristics, also reflects that the univariate analysis performed for gender, co-morbidities, alcohol/smoking, and education status with adverse events showed an association. However, by multiple logistic regression analyses, it was found that none of the variables was associated with an adverse event.

**Table 1:** Demographic characteristics. DM: Diabetes mellitus; HTN: Hypertension; DSTB: Drug-sensitive tuberculosis; MDR: Multidrug-resistant; XDR: Extensively drug-resistant. The missing values are excluded from the analysis. p <0.05 is considered significant.

Parameters		Without adverse events (n = 127)	With adverse events (n = 92)	P-value
		n (%)	n (%)	
Age in years (mean ± SD)		46.13 ± 14.89	45.48 ± 17.884	0.112
BMI kg/m2 (mean ± SD)		20.96 ± 4.88	19.45 ± 5.36	0.776
Gender	Male	90 (70.9)	52 (56.5)	0.028*
	Female	37 (29.1)	40 (43.5)	
Comorbidity	DM	21 (16.5)	26 (28.3)	0.000**
	HTN	4 (3.1)	5 (5.4)	
	DM and HTN	1 (0.8)	0	
	Cardiac	0	1 (1.1)	
	Hypothyroidism	0	1 (1.1)	
	No comorbidity	47 (37)	46 (50)	
Habits	Alcohol	8 (6.3)	15 (16.3)	0.000**
	Smoking	4 (3.1)	2 (2.2)	
	Alcohol and Smoking	35 (27.6)	18 (19.6)	
	Tobacco	1 (0.8)	3 (3.3)	
	No other	41 (32.3)	50 (54.3)	
Type of TB	Pulmonary TB	93 (73.2)	61 (66.3)	0.268
	Extrapulmonary TB	34 (26.8)	31 (33.7)	
	DSTB	121 (95.3)	81 (88)	
Type of regimen	All-oral longer MDR	1 (0.8)	1 (1)	
	Shorter MDR	0	2 (2.2)	
	Isoniazid-monoresistant	4 (3.1)	5 (5.4)	
	XDR	1 (0.8)	3 (3.3)	
Education	Illiterate	16 (12.6)	26 (28.3)	0.000*
	Primary	11 (8.7)	7 (7.6)	
	Middle	32 (25.2)	23 (25)	
	Higher	8 (6.3)	8 (8.7)	
	Graduation/Diploma	13 (10.2)	6 (6.5)	
	Postgraduate	1 (0.8)	12 (13)	

The comparison of the type of regimen with the severity of the adverse event is represented in Table 2.

**Table 2:** Comparison of type of regimen with severity of adverse events. N*: Number of patients*; DSTB: Drug-sensitive tuberculosis; DRTB: Drug-resistant tuberculosis. The missing values are excluded.

Type of regimen	Severity (n)*			P-value
	Mild	Moderate	Severe	
DSTB	32	28	15	0.626
DRTB	2	4	2	

It was observed that among the patients on the DSTB vs. DRTB regimen, 60 and 6 patients, respectively, experienced adverse events, which were mild to moderate in nature, and 15 and 2, respectively, had severe form. However, the data on severity was missing for nine patients from among the total of 92 patients with adverse events.

**Figure 1:**
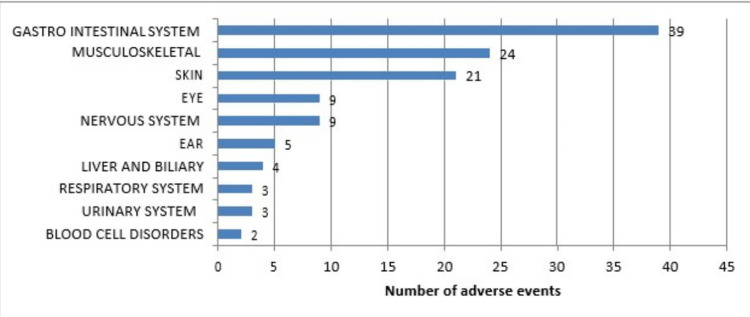
WHO System Organ Classes of adverse events.

The adverse events noted were grouped based on the system involved and represented using the WHO system organ classes in Figure 1. The GI tract (GIT) was the most common body system involved (39), followed by the musculoskeletal system and skin disorders (24 and 21), respectively. The sub-classes of adverse events within each system involved are represented in Table 3, in which vomiting (24) was the most common presentation in TB patients, followed by joint pain (19) and skin rashes (14).

**Table 3:** Distribution of adverse events in tuberculosis patients on ATT. ATT: Antitubercular therapy.

WHO System Organ Classes (n)	Adverse Events
GI disorders	Vomiting (24), diarrhea (2), nausea (3), gastritis (5), abdominal pain (4), and oral thrush (1)
Musculoskeletal system disorders	Joint pain (19), leg swelling (1), back pain (4), and generalised body pain (1)
Skin disorders	Rashes (14), itching (5), skin discoloration (1), hyperpigmented pustules (1)
Eye	Blurred vision (8), optic neuritis (1)
Nervous system	Giddiness (4), headache (1), peripheral neuropathy (1), seizure (1), fever (1), and depression (1)
Ear	Hearing loss (5)
Liver and biliary disorders	Liver enzymes increased (3), jaundice (1)
Respiratory system	Breathing difficulty (3)
Urinary system	Urine discoloration (3)
Blood cell disorders	Thrombocytopenia (1), anemia (1)

The onset, severity, and outcome of adverse events are shown in Table 4. Most of the adverse events were latent (60.9%), followed by sub-acute (28.3%) and acute (6.5%) onsets. They were categorized as acute if the event occurred within 60 minutes of drug administration, subacute and latent if the event occurs within 1-24 hours and one day to several weeks, respectively. Among the adverse events reported, most were mild to moderate (71.8%), which required supportive management or dose adjustments for a short duration, followed by severe, which required hospitalization or intervention to prevent permanent damage (18.5%).

**Table 4:** Onset, severity, and outcome of adverse events. n*: Number of patients; ADR: Adverse drug reaction. The missing values are excluded.

Parameters		n*	%
Onset of ADRs	Acute	6	6.5
	Sub-acute	26	28.3
	Late-onset	56	60.9
Severity	Mild	34	37.0
	Moderate	32	34.8
	Severe	17	18.5
Causality assessment	Definite	23	25
	Probable	38	41.3
	Possible	19	20.7
	Unlikely	2	2.2

As per Naranjo's probability scale for causality of the events, most of the events were probable in nature (41.3%), definite and possible, with 25% and 20.7%, respectively. The information about the adverse event recorded was as per the standard operative procedure of the Indian Pharmacopoeia Commission on the suspected adverse reporting form.

## Discussion

This study was planned to estimate the frequency of ADRs to ATT and associated risk factors among TB patients. In this study, among the 219 TB patients started on ATT (first-line/second-line drugs), 42% had adverse events.

The patients receiving ATT with adverse events had a mean age of 45.48 ± 17.884, according to the study of Ramanath KV and Ramesh S [15]. However, the study by Chhetri AK et al. reported that for ADRs for the first-line ATT, the patients were 21-30 years of age [16]. In the present study, ADRs were found to be more in females. The females were found to have 1.8 times the risk than males to develop the adverse events to TB drug, which was also observed by Shareef J et al. [17]. In this study, most of the patients were literate. Though this may not directly relate to adverse events, this can increase the identification and reporting of the event to the healthcare facility.

The GI system was the most common system involved in adverse events comprising vomiting, gastritis, and abdominal pain. This was consistent with Tak DK et al., except that the patients had more epigastric pain and loss of appetite [18].

The next most common system which showed adverse events was the musculoskeletal system, where the patients had joint pain, low back pain, or leg swelling. These observations were similar to Ramanath KV, Ramesh S, and Koju D et al., who have also reported common musculoskeletal system involvement in the form of myalgia [15,19].

The skin involvement comprising mainly of rashes and itching was seen in almost 23% of the patients. This was in agreement with the findings of Ramanath KV and Ramesh S, in which they observed a 27.34% of dermatological system involvement. However, other studies have shown a reporting of 5- 10% [10,20]. The liver enzymes were affected in three of the patients in our study. The most likely drug causing deranged liver function test was ethambutol in two of the cases in our study, as ascertained by Naranjo’s scale. A rechallenge was done for both of these patients. A 20-year-old female patient, while on an isoniazid-mono resistant regimen, developed anemia, which was attributed to linezolid. A study by Hanai Y et al. had described the factors for linezolid-induced anemia [21]. In one of the studies on ADRs to TB drugs, anemia accounted for 2.7% of the adverse events [10].

A total of 60.9% of adverse events experienced by TB patients on ATT was predominantly late onset in nature. Tak DK et al., in their study, reported 33.33% of the adverse events to be latent [18], and in another study, it was 74.3% [10]. The majority of adverse events were self-limiting and did not require discontinuation of the ATT regimen. Furthermore, most of the adverse events reported in our study were "probable" as per the Naranjo algorithm, which was also observed in a study conducted in Bihar [20].

This study showed a prevalence of adverse events in the present study as 42%. This may be attributed to drug-drug interactions, cumulative toxicity, or maybe due to genetic influence, which needs to be validated. The limitation of the study was the short duration of the study period, which might impact the actual volume of the problem. Despite these limitations, we could identify ADRs like hepatotoxicity, peripheral neuropathy, optic neuritis, and depression which add to the current literature regarding the adverse events of anti-TB drugs. However, considering the availability of resources and advancement in science, we could minimize this impact of ADRs, though it could not be completely eliminated. We had reported these adverse events to ATT from the present study to the regional pharmacovigilance center in Puducherry. We submitted a report to the state or committee for their further necessary action.

## Conclusions

The current study shows the frequency of adverse events in patients receiving ATT. The females were found to be at more risk than males for adverse events. It was found that the GI system was most affected as a known reaction to TB therapy followed by the musculoskeletal system. With more effective pharmacovigilance measures implementation, the adverse events being one of the factors for treatment interruption can be overcome.

## References

[1] (2019). WHO: Global tuberculosis report 2019. https://www.who.int/publications/i/item/9789241565714.

[2] (2020). India Tuberculosis Report 2020. https://tbcindia.gov.in/WriteReadData/l892s/India%20TB%20Report%202020.pdf.

[3] (2017). Guidelines for programmatic management of drug-resistant tuberculosis in India. https://tbcindia.gov.in/index1.php?lang=1&level=2&sublinkid=4780&lid=3306.

[4] Sadiq S, Khajuria V, Tandon VR, Mahajan A, Singh JB (2015). Adverse drug reaction profile in patients on anti-tubercular treatment alone and in combination with highly active antiretroviral therapy. J Clin Diagn Res.

[5] Wu S, Zhang Y, Sun F, Chen M, Zhou L, Wang N, Zhan S (2016). Adverse events associated with the treatment of multidrug-resistant tuberculosis: a systematic review and meta-analysis. Am J Ther.

[6] Diallo T, Adjobimey M, Ruslami R (2018). Safety and side effects of rifampin versus isoniazid in children. N Engl J Med.

[7] Yates TA, Nunn AJ (2016). Efficacy and safety of regimens for drug-resistant tuberculosis. Lancet Infect Dis.

[8] Dela AI, Tank NK, Singh AP, Piparva KG (2017). Adverse drug reactions and treatment outcome analysis of DOTS-plus therapy of MDR-TB patients at district tuberculosis centre: a four year retrospective study. Lung India.

[9] Prasad R, Singh A, Gupta N (2021). Adverse drug reactions with first-line and second-line drugs in treatment of tuberculosis. Ann Natl Acad Med Sci (India).

[10] Dhanalakshmi D, MounaReddy V, Anusha K, Vikas K, Kumar KA (2017). A study on adverse drug reactions in tuberculosis patients maintained on DOTS protocol in government chest & TB hospital Warangal district. IOSR-JDMS.

[11] Sood A, Bansal R, Sharma A, Himani H, Bhagra S, Kansal D (2016). Profile of adverse drug reactions in patients on anti-tubercular drugs in a sub Himalayan rural tertiary care teaching hospital. Int J Res Med Sci.

[12] Naranjo CA, Busto U, Sellers EM (1981). A method for estimating the probability of adverse drug reactions. Clin Pharmacol Ther.

[13] Hartwig SC, Siegel J, Schneider PJ (1992). Preventability and severity assessment in reporting adverse drug reactions. Am J Hosp Pharm.

[14] Hoigné R, Jaeger MD, Wymann R (1990). Time pattern of allergic reactions to drugs. Agents Actions Suppl.

[15] Ramanath KV, Ramesh S (2012). A study on assessment of adverse drug reactions in tuberculosis patients. Am J Pharm Tech Res.

[16] Chhetri AK, Saha A, Verma SC, Palaian S, Mishra P, Shankar PR (2008). A study of adverse drug reactions caused by first line anti-tubercular drugs used in Directly Observed Treatment, Short course (DOTS) therapy in western Nepal, Pokhara. J Pak Med Assoc.

[17] Shareef J, Nandakumar UP, Bhat M (2018). A study on assessment of adverse drug reactions in patients with tuberculosis in a tertiary care teaching hospital. J Appl Pharm Sci.

[18] Tak DK, Acharya LD, Gowrinath K, Rao Padma GM, Subish P (2009). Safety evaluation of antitubercular therapy under Revised National Tuberculosis Control Programme in India. J Clin Diagn Res.

[19] Koju D, Rao BS, Shrestha B, Shakya R, Makaju R (2005). Occurrence of side effects from anti-tuberculosis drugs in urban Nepalese population under dots treatment. Kathmandu Univ Med J.

[20] Mogali SM, Ratnakar JS, Kotinatot BC (2020). Study of adverse drug reactions among tuberculosis patients in a tertiary care hospital: a retrospective observational study. Int J Basic Clin Pharmacol.

[21] Hanai Y, Matsuo K, Ogawa M (2016). A retrospective study of the risk factors for linezolid-induced thrombocytopenia and anemia. J Infect Chemother.

